# Metallurgical investigation of wire breakage of tyre bead grade

**DOI:** 10.1016/j.csefa.2015.09.003

**Published:** 2015-10-13

**Authors:** Piyas Palit, Souvik Das, Jitendra Mathur

**Affiliations:** R&D and Scientific Services, Tata Steel Limited, Jamshedpur 831 001, India

**Keywords:** Tyre bead grade, Button like defect, Surface martensite

## Abstract

Tyre bead grade wire is used for tyre making application. The wire is used as reinforcement inside the polymer of tyre. The wire is available in different size/section such as 1.6–0.80 mm thin Cu coated wire. During tyre making operation at tyre manufacturer company, wire failed frequently. In this present study, different broken/defective wire samples were collected from wire mill for detailed investigation of the defect. The natures of the defects were localized and similar in nature. The fracture surface was of finger nail type. Crow feet like defects including button like surface abnormalities were also observed on the broken wire samples. The defect was studied at different directions under microscope. Different advanced metallographic techniques have been used for detail investigation. The analysis revealed that, white layer of surface martensite was formed and it caused the final breakage of wire. In this present study we have also discussed about the possible reason for the formation of such kind of surface martensite (hard-phase).

## Highlights

• Tyre bead grade wire is used for tyre making application.
• During tyre making operation at tyre manufacturer company, wire failed frequently in brittle mode.
• Crow feet like defects including button like surface abnormalities were also observed.
• The analysis revealed that, surface martensite was formed and it caused the final breakage.

## 1 Introduction

**Fig. 1:**
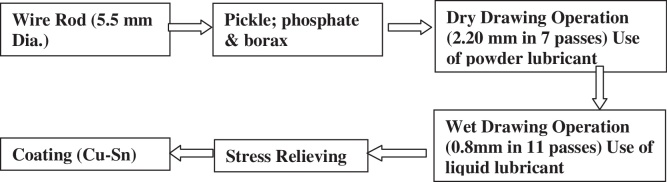
Flow diagram of wire rod to wire drawing process.

Tyre bead grade with Cu-coating was conventionally used for tyre making application [1]. During tyre making operation at tyre manufacturer company, wire failed frequently during bending operation at brittle manner. During bending operation such kind of breaking was also happened at wire mill. Different breakages as well as defective samples have been collected from different coils. The wire manufactured by drawing process from 5.5 mm wire rod [2], [3]. Two stage of drawing process is involved to making of final wire. After the drawing operation stress reliving and Cu–Sn coating of wire was carried. The process details are mentioned in Fig. 1.

## 2 Visual observation

**Figs. 2–5:**
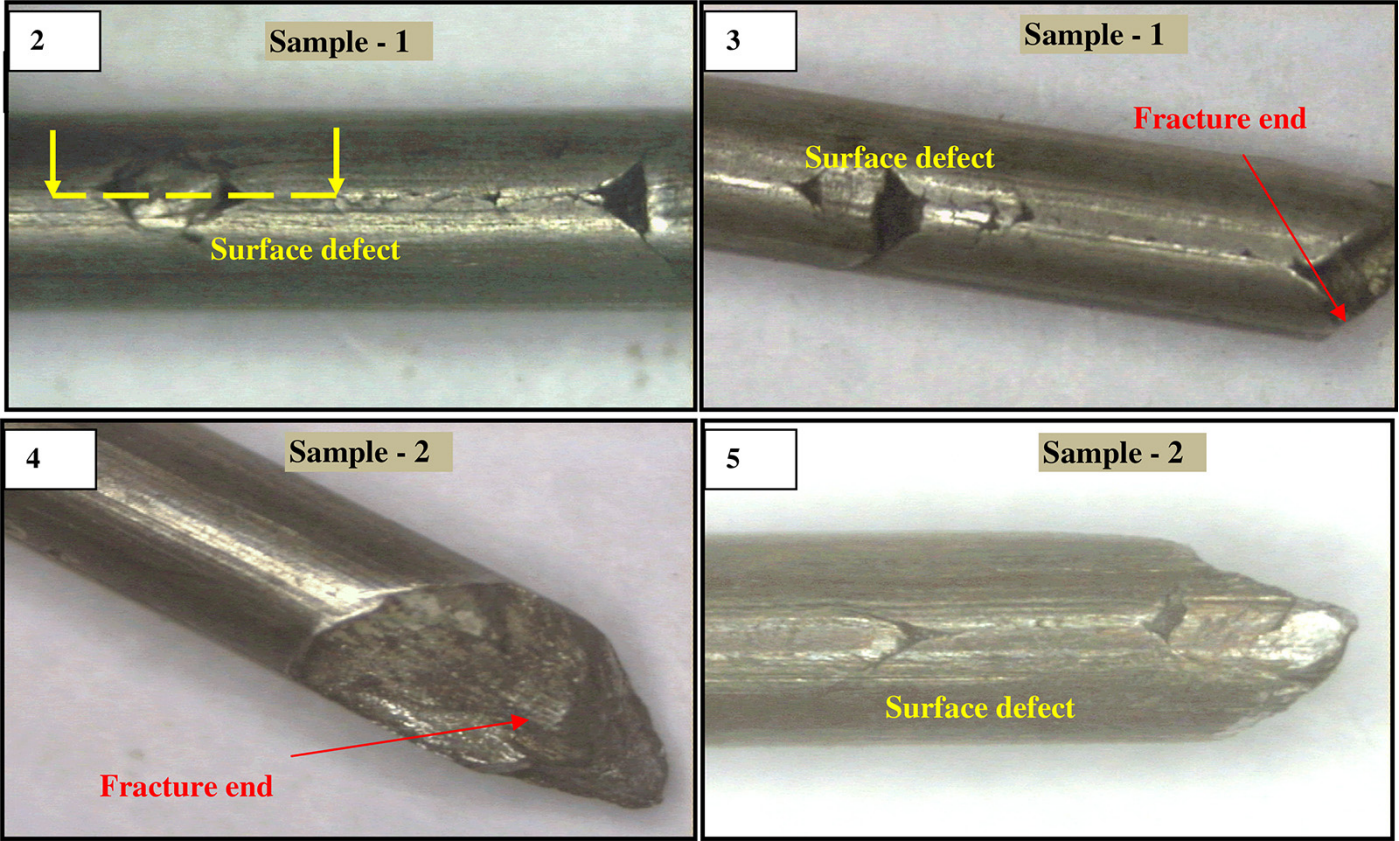
(2, 3) Closer view of surface defects of failed wire samples #1. (4, 5) Closer view of surface defects of failed wire samples #2.

Two pieces of breakage wire samples were collected from the drawing mill for investigations. The samples were cleaned with acetone to remove dirt for visual examination prior to metallographic sample preparation. Visual examination is carried out in stereoscope. Surface appearance of the defects in all wire samples was of similar in nature. The fracture surface revealed finger nail type (Figs. 4 and 5). Crow feet like defects including button like surface abnormalities were observed on the broken wire samples. The defect was observed near the fracture end and which was very much localized in nature (Figs. 2 and 3).

## 3 Chemical analysis

Chemical analysis of wire samples was carried out using combustion infrared technique (LECO, TC600) for carbon and sulphur contents. An inductively coupled plasma atomic emission spectroscopy (ICP-AES) instrument was used to determine amounts of rest of the elements. The chemistry of wire sample confirmed to high carbon steel grade (C-70). Chemical analysis result is presented in Table 1.

## 4 Metallography analysis

### 4.1 Microstructural analysis

**Figs. 6 and 7:**
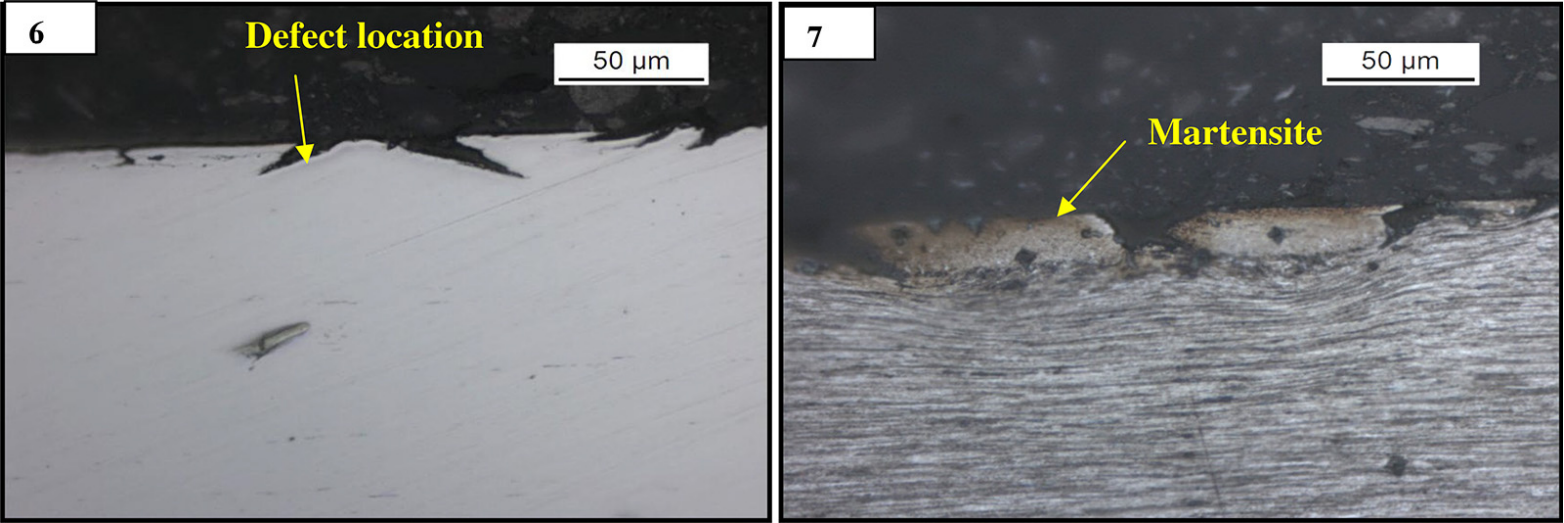
(6) Un-etched micrograph of the cross section of the defect location in longitudinal micro specimen. (7) Etched microstructure of the same.

**Figs. 8–11:**
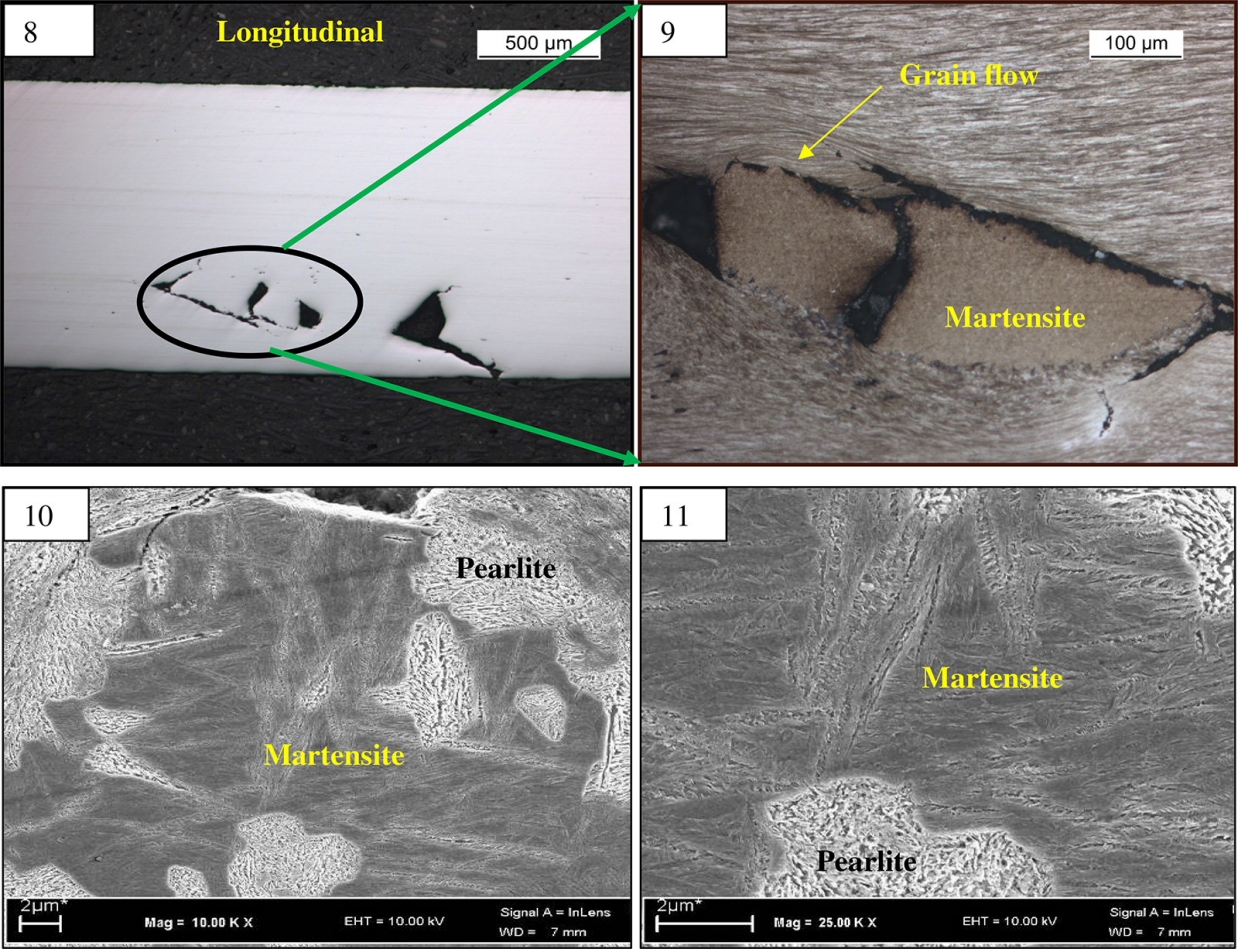
(8) Un-etched micrograph of the top view of the defect in longitudinal micro specimen at 50× magnification. (9) Etched microstructure of the same at 200× magnification revealed martensite at the defect location. (Top view of the defect; etched after marginal polishing of surface.) (10, 11) Martensite at higher magnification under SEM.

Micro specimens were prepared from the fractured end as well as defect location of wire samples for conducting light optical microscopic examination and scanning electron microscopy (SEM). These samples were individually mounted in conductive mounting and polished by conventional metallographic techniques for scratch free surface. The polished samples were etched in 3% nital solution (3 mL HNO_3_ in 97 mL ethyl alcohol), and both un-etched and etched samples were examined in a light microscope to observe microstructural constituents. Un-etched sample shows surface defect in longitudinal as well in transverses direction (Figs. 6 and 7, Figs. 8–11). Etched microstructure of the longitudinal samples revealed presence of brown layer near the defect location. The thickness of the brown layer is around 30–40 μm (Fig. 7). From microstructure analysis the brown layer appeared to be of martensite (which was further verified by micro hardness value and SEM analysis; Table 2). Severe grain flow was observed along the defect location. The microstructure of the matrix revealed cold drawn pearlite structure (Figs. 9–11).

### 4.2 Micro hardness test

**Fig. 12:**
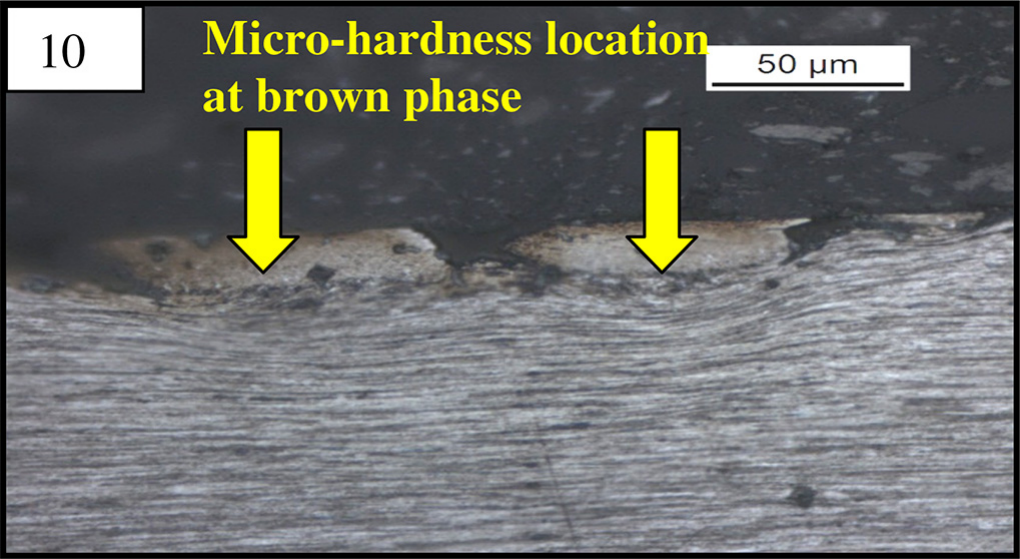
Etched microstructure of the defect location showing indentation in the brown layer (surface martensite) and the drawn pearlite matrix.

The micro hardness of different phases observed in the broken wire samples was determined in a pneumatically controlled automatic micro hardness tester (Leco-LM247AT). An applied load of 50 gf was used during testing, and several indentations were made to determine the hardness of different phases (Fig. 12). The average hardness of the matrix is about 461 HV, and the average hardness value of the brown phase is about 624 HV (Table 2).

### 4.3 EDS analysis

**Fig. 13:**
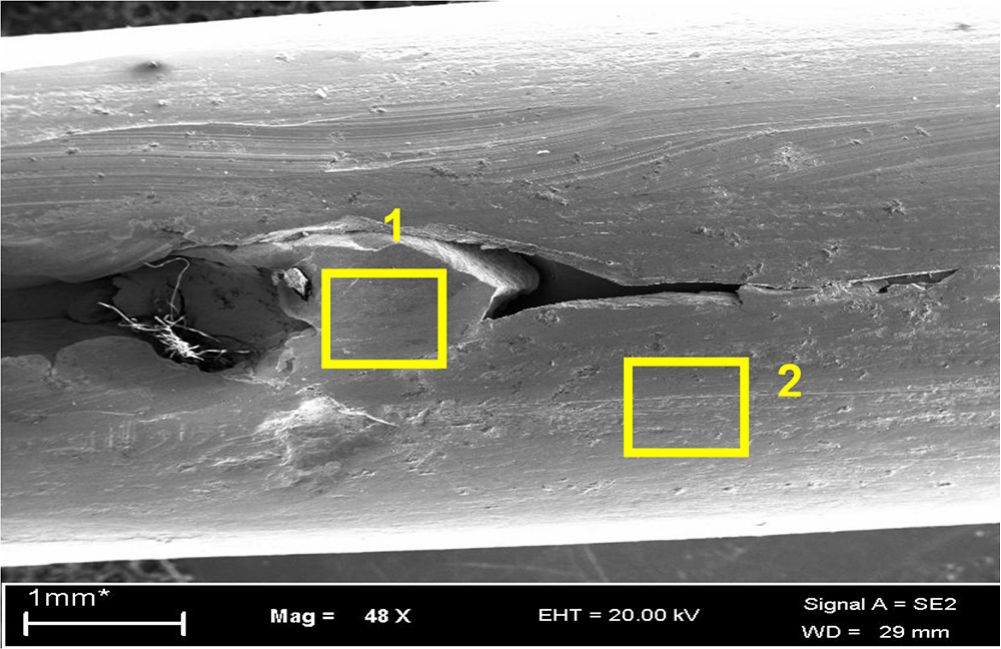
SEM image of defect sample in as received condition.

EDS analysis was carried out in the as received sample to find out the elemental difference between parent and the defect region (Fig. 13). EDS analysis reveals presence of tungsten (W) in the martensite region in concentration of more than 2% as shown in Table 3. As the element is not contained in bulk, which indicates that the material transfers between the mating bodies i.e. an intense adhesive sliding wear. Other elements remains almost constant indicate that martensitic transformation took place due to thermal effect with rapid quenching of local austenite produced by friction [4].

## 5 Discussions

Premature wire failures were observed during bending operation before tyre making process. The nature of the defects was of similar type in all the failed samples. The fracture surface was of finger nail type. Crow feet like defects including button like surface abnormalities were observed on the broken wire samples. The surface defect was observed near the fracture end and in localized manner. Etched microstructure of the longitudinal samples revealed presence of brown layer near the defect location. The thickness of the brown layer is around 30–40 μm. From microstructure analysis the brown layer appeared to be of martensite (which was further verified by micro hardness value and SEM analysis). Severe grain flow was observed along the defect location. The microstructure of the matrix revealed cold drawn pearlite structure. The average hardness of the matrix is about 460 HV, and the average hardness value of the brown phase is around 650 HV. This type of layer is generated during wire drawing due to lack of lubrication as no segregation was observed [4]. The martensite layer which forms a brown layer in the surface is very brittle in nature (high hardness). This surface martensite helps to propagate cracks from the pearlite–martensite interface and which leads to failure during drawing or its successive operations. The martensite formed in the surface is a thermal phenomenon generated during friction causes surface temperature rise followed by rapid cooling due to mass effect of bulk. EDS analysis reveals presence of tungsten (W) in the martensite region in concentration of more than 2% as the element is not contained in bulk, which indicates that the material transfers between the mating bodies i.e. an intense adhesive sliding wear. The martensite formed in the surface was generated during drawing process probably due to lack of localize lubrication [5]. Due to improper lubrication, during drawing of high carbon wires sometimes temperature reaches up to austenitic range due to heat generated, because of plastic deformation and friction between wire and die [6].

## 6 Conclusion

Presence of martensite (hard phase) in the surface of the wire samples caused breakage during drawing. It could be envisaged from the surface characteristics and microstructure vis-à-vis the occurrence of failure that the hard-phase generated during drawing due to improper lubrication.

## Figures and Tables

**Table 1:** Chemical analysis result of wire samples (wt.%).

Spec	Chemistry							Section (mm)
	C	Mn	S	P	Si	Cr	N_2_ (ppm)	
Wire sample 1	0.69	0.66	0.012	0.019	0.18	0.01	33	1.6
Wire sample 2	0.71	0.69	0.013	0.022	0.21	0.01	41	0.8

**Table 2:** Micro hardness test result.

Sample no.	Parent phase (HV 50 gf)	Brown phase (HV 50 gf)
1	470,460	667,663
2	452,462	660,625

**Table 3:** EDS analysis.

Location	Si	Mn	W	Fe
Martensite (1)	0.30	0.87	2.19	96.64
Matrix (2)	0.38	0.91	–	98.71
